# Validation and optimisation of a touchscreen progressive ratio test of motivation in male rats

**DOI:** 10.1007/s00213-018-4969-6

**Published:** 2018-07-14

**Authors:** Jonathan M. Hailwood, Christopher J. Heath, Trevor W. Robbins, Lisa M. Saksida, Timothy J. Bussey

**Affiliations:** 1 Department of Psychology and Behavioural and Clinical Neuroscience Institute, University of Cambridge, Downing Street, Cambridge, CB2 3EB UK; 2 School of Life, Health and Chemical Sciences, The Open University, Walton Hall, Milton Keynes, MK7 6AA UK; 3 Molecular Medicine Research Group, Robarts Research Institute & Department of Physiology and Pharmacology, Schulich School of Medicine & Dentistry, Western University, London, ON Canada; 4 The Brain and Mind Institute, Western University, London, ON Canada

**Keywords:** Progressive ratio schedule, Touchscreen, Motivation, Rat

## Abstract

**Rationale:**

Across species, effort-related motivation can be assessed by testing behaviour under a progressive ratio (PR) schedule of reinforcement. However, to date, PR tasks for rodents have been available using traditional operant response systems only.

**Objectives:**

Touchscreen operant response systems allow the assessment of behaviour in laboratory rodents, using tasks that share high face validity with the computerised assessments used in humans. Here, we sought to optimise a rat touchscreen variant of PR and validate it by assessing the effects of a number of manipulations known to affect PR performance in non-touchscreen paradigms.

**Methods:**

Separate groups of male Sprague-Dawley rats were trained on PR schedules with either linear (PR4) or exponential (PREXP) schedules of reinforcement. PR performance was assessed in response to manipulations in reward outcome. Animals were tested under conditions of increased reward magnitude and following reward devaluation through a prefeeding procedure. Subsequently, the effects of systemic administration of the dopamine D2/D3 receptor antagonist raclopride and the psychostimulant d-amphetamine were examined as traditional pharmacological methods for manipulating motivation.

**Results:**

Rats reinforced under PR4 and PREXP schedules consistently showed differential patterns of response rates within sessions. Furthermore, both PR4 and PREXP schedules were sensitive to suppression by prefeeding or raclopride administration. Performance under both schedules was facilitated by increasing reward magnitude or *d*-amphetamine administration.

**Conclusions:**

Taken together, these findings mirror those observed in lever-based PR paradigms in rats. This study therefore demonstrates the successful validation of the rat touchscreen PR task. This will allow for the assessment of motivation in rats, within the same touchscreen apparatus used for the assessment of complex cognitive processes in this species.

## Introduction

Impaired motivated behaviour represents an unmet clinical need in a number of neuropsychiatric and neurodegenerative disorders. Such impairment, often referred to as ‘apathy’, are a common and debilitating symptom in disorders such as schizophrenia (Foussias et al. 2014), major depression (Treadway and Zald 2011), Alzheimer’s disease (Landes et al. 2001), Parkinson’s disease (Pedersen et al. 2009) and Huntington’s’ disease (Naarding et al. 2009). Across disorders, apathy can severely affect patients’ quality of life (Ho et al. 1998; Boyle et al. 2003; Starkstein et al. 2006; Aarsland et al. 2007) and has been linked to accelerated disease progression and increased mortality rates (Starkstein et al. 2006; Spalletta et al. 2015). Standard treatment approaches for these disorders have little impact upon apathy (Fervaha et al. 2015; Lanctôt et al. 2017), highlighting the need for novel pharmacological targets. A key stage of developing novel treatments typically involves displaying the effectiveness of a compound in a preclinical rodent model. Therefore, the ability to measure motivated behaviours in rodents is of crucial importance.

Motivated behaviour can be divided into activational and directional components (Robbins and Everitt 1982; Salamone 1988). Directional processes allow behaviour to be directed towards appetitive and away from aversive stimuli. Activational aspects of motivation allow organisms to overcome costs or obstacles that are associated with obtaining goals (Salamone 1988). In a number of disorders associated with motivational impairments, activational processes appear disrupted (Barch et al. 2014; Chong et al. 2015; Salamone et al. 2016). Activational components of motivated behaviour can be probed in the laboratory through studying the exertion of effort. One widely used assay involves studying behaviour under a progressive ratio (PR) schedule of reinforcement (Hodos 1961). This task probes the ability of an organism to maintain instrumental responding (such as lever pressing or nose-poking) under increasing work demands. As the response requirement increases, an animal will eventually cease responding. The amount of effort an animal is willing to expend in pursuit of appetitive reinforcement, expressed as the maximum number of responses to obtain a single reward, is referred to as the breakpoint (BP, Stewart 1975). PR schedules have been used to study effort exertion across a number of species including rats (Hodos 1961); mice (Randt and Quartermain 1972); pigeons (Dardano and Sauerbrunn 1964); nonhuman primates (Griffiths et al. 1975) and humans (Roane et al. 2001).

One recent refinement in preclinical animal testing has been the development of touchscreen operant response systems (Bussey et al. 2012; Hvoslef-Eide et al. 2015). These systems allow the assessment of a number of cognitive domains including attentional processes and long-term and working memory (Horner et al. 2013; Mar et al. 2013; Oomen et al. 2013) within a single environment. These systems also allow for the use of assays that share a high degree of face validity with the automated computerised testing batteries increasingly used in clinical populations (Sahakian and Owen 1992; Barnett et al. 2010; Bland et al. 2016) and nonhuman primates (Weed et al. 1999). Although face validity does not guarantee construct validity, it may help facilitate cross-species translation of results. Previous research has shown that, similar to lever and nose-poke manipulanda, rodent touchscreens can support the sustained repetitive response behaviour required in ratio schedules such as PR, and that this schedule can be successfully implemented in mice using the touchscreen system (Heath et al. 2015). The development of a validated rat touchscreen PR test would allow the assessment of motivation in the rat using the same reinforcers, responses and test setting as those used in the assessment of other complex behavioural constructs in the same apparatus. This would allow motivated behaviour to be assessed alongside and in a comparable way to other cognitive processes as part of a battery approach in situations where the rat is the favoured species. In spite of general consistency between touchscreen-based assays and traditional lever-based or nose-poke systems (cf Humby et al. 1999; Romberg et al. 2013), there have been reports of differential sensitivity to pharmacological manipulations in mice (see Heath et al. 2015). Therefore, it is necessary to verify the sensitivity of the touchscreen-based PR task in rats to manipulations previously shown to affect performance.

PR tasks can vary in the nature of the schedule of reinforcement used. Some PR schedules increase in a linear fashion (e.g. Skjoldager et al. 1993; Aberman et al. 1998; Bensadoun et al. 2004; Heath et al. 2015), whereas others employ exponentially increasing ratios (e.g. Poncelet et al. 1983; Mobini et al. 2000; Rickard et al. 2009). It is not known whether manipulations that affect PR performance differentially affect behaviour reinforced under these different schedule types. We therefore assessed performance on two separate reinforcement schedules: the linear PR4 and the exponential PREXP schedules. We then sought to determine how these schedules were affected by a number of manipulations that have been previously been shown to affect performance. Initially, we modulated the reward outcome value. Firstly, this was achieved by increasing the magnitude of reward, which was hypothesised, based on previous reports, to increase breakpoint (Skjoldager et al. 1993; Eagle et al. 1999; Rickard et al. 2009). Secondly, the reinforcer was devalued through a prefeeding procedure, which was predicted to decrease breakpoints (Skjoldager et al. 1993; Eagle et al. 1999). Subsequently, performance was assessed following systemic administration of dopaminergic compounds. Based on previous reports, it was predicted that administration of the D2/D3 receptor antagonist raclopride would disrupt PR performance (Cheeta et al. 1995; Aberman et al. 1998). Finally, it was predicted that PR performance would be facilitated following systemic d-amphetamine administration (Poncelet et al. 1983; Mobini et al. 2000; Bensadoun et al. 2004).

## Methods

### Animals

Twenty-four male Sprague-Dawley rats (Charles River, UK) were used in the current experiment. Animals were group housed (four per cage) in a light- and temperature-controlled environment (lights on 1900-0700). All testing took place in the animals’ dark cycle. Following at least 7 days habituation to the facility, animals were placed on a programme of controlled feeding and maintained at no less than 85% of their free feeding body weight. All experiments were regulated under the Animals (Scientific Procedures) Act 1986 Amendment Regulations 2012 and following ethical review by the University of Cambridge Animal Welfare and Ethical Review Body (AWERB).

### Apparatus

All testing took place within automated rat touchscreen operant conditioning chambers (Campden Instruments Ltd., Loughborough, UK) described in detail previously (Horner et al. 2013). The operant chambers consisted of black plastic walls in a trapezoidal shape (height 30 cm, length 33 cm, width 25 cm at screen, 13 cm at magazine). The operant chambers were contained within light and sound-attenuating boxes. Each operant chamber was fitted with a 38.1-cm touch-sensitive LCD screen. Each screen was equipped with infrared (IR) beams positioned less than 5 mm away from the screen, which detected responses without requiring any force to be applied to the screen itself. On the opposing side was a magazine connected to a pellet dispenser that delivered standard 45 mg dustless pellets (TestDiet, Indiana, USA). The food tray was fitted with a light and an IR beam that registered magazine entries. Front and rear IR beams were fitted to monitor the rats’ activity within the operant chamber. Black plastic masks were fitted to the touchscreens that had five 9 cm^2^ square response apertures, spaced 1 cm apart.

### Pretraining

Behavioural testing consisted of one session per day (5–7 days per week). All animals were initially given a 20-min habituation session. During this session, the boxes were active but no stimuli were presented. Following this, rats underwent 1 day of screen press training. A white square stimulus was presented in the central aperture for 30 s. A single response to this stimulus resulted in three food pellets being delivered. Stimulus offset and a short tone (1000 ms, 3 kHz) accompanied reward delivery. Following a 5-s inter-trial interval (ITI) the stimulus returned to the screen. If no response was made within 30 s, the trial ended and a single food pellet was delivered, accompanied by stimulus offset and the tone. Each session was terminated following 100 rewards being delivered or 45 min having elapsed.

### Fixed ratio training

Rats then underwent fixed ratio (FR) 1 training. During these sessions, a single response to the central stimulus was required for a single pellet reward delivery. Reward delivery was again accompanied by the tone. A 5-s inter-trial interval (ITI) was employed. Each session was terminated following 45 min or 100 trials being completed. All animals were required to complete 100 trials within the 45 min before moving on to the next stage of training. The subsequent training stage consisted of FR5 responding, where five responses were required for each reward delivery. The first four responses in a trial were accompanied with a shorter ‘click’ tone (10 ms, 3 kHz) and a brief (500 ms) stimulus offset. The stimulus offset and brief ‘click’ tone were added to provide audio-visual feedback to the rat of a successful stimulus response. The fifth response to the stimulus completed the trial and resulted in delivery of reward and the longer duration tone. All other parameters were identical to the FR1 stage of training. Each session was terminated following 100 trials (i.e. 500 target responses) or after 45 min. Each animal was required to complete 100 trials within a session before being placed on a PR schedule of reinforcement.

### Progressive ratio

Animals were randomly assigned to either a linear (PR4) or exponential (PREXP) schedule (*n* = 12 each). The PREXP schedule chosen is commonly used in research, whereas the PR4 schedule is that used in the mouse touchscreen equivalent that can stably support behaviour at a level that can be bi-directionally manipulated by pharmacological interventions in touchscreens (Heath et al. 2015). On both schedules, the number of target responses required increased following completion of each trial. On the linear schedule, the response requirement began at one and increased by four on each subsequent trial (yielding response requirements of 1, 5, 9, 13, 17 etc.). The exponential schedule increased according to the formula (5 * e(0.2*n)- 5), where *n* is the trial number, yielding response requirements of 1, 2, 4, 6, 9, 12 etc., to the nearest whole number. If no response was made to the touchscreen within 180 s, on either schedule, the session was terminated (based upon previous reports, Wirtshafter and Stratford 2010; Klinkenberg and Blokland 2011; Enkel et al. 2014); otherwise, sessions ended after 45 min elapsing.

### Outcome manipulations

Outcome manipulation probes were delivered in a within-subject cross-over design. Firstly, rats underwent a reward magnitude probe. On these days, rats received either a standard (single pellet) or an increased (three pellet) reward following each completed ratio. The groups were counterbalanced so that on each day equal numbers of PR4 and PREXP rats were in each condition. A baseline day was administered between test days, where rats were tested as normal and received a single pellet reward for each completed trial. On the prefeeding probe days, rats were randomly assigned to a prefeed or no prefeed (control) condition. Rats within the prefeed condition were given 1 h of free access to homecage lab chow prior to testing. Rats within the no prefeed control condition were tested as normal with chow provided after the PR session was completed. Equal numbers of PR4 and PREXP rats were tested on both conditions on each test day. Again, a baseline day was given between test days to ensure no carry-on effects of prefeeding were observed upon PR performance.

### Dopaminergic manipulations

Pharmacological challenges were delivered in a within-subject Latin square design. All drugs were dissolved in physiological saline and delivered via intraperitoneal injections at a volume of 1 ml/kg of each rat’s body weight, 30 min prior to PR testing. Rats were returned to their home cages for the post injection period of 30 min. The D2/D3 receptor antagonist s(−)raclopride(+)-tartrate salt (Sigma-Aldrich, Dorset, UK) was administered at doses of 0, 0.03 and 0.3 mg/kg. Following a 7-day washout period, d-amphetamine sulphate (Sigma-Aldrich, Dorset, UK) was administered at doses of 0, 0.1 and 1 mg/kg.

### Behavioural measures

The primary measure of interest was breakpoint (BP), defined as the number of target responses made in the last successfully completed trial for each subject. The mean post reinforcement pause (PRP), defined as the latency between an animal removing its head from the magazine following reinforcement and the first touchscreen target response of the subsequent trial, was also assessed. The total number of responses made for each reward earned was calculated from the total number of touchscreen responses (therefore, including those made in incomplete trials) Response rates were analysed as previously described (Simpson et al. 2011; Phillips et al. 2017). Briefly, response rates per trial were calculated by dividing the number of responses made in each trial by the time taken to complete each trial, from the first response (therefore, excluding post reinforcement pauses). The first two trials in each session were excluded from the response rate analyses. The first trial was excluded as it only involved a single lever press, meaning it is not possible to calculate a response rate. The second trial was excluded as it only required two responses in the PREXP schedule. The low number of responses needed in this condition may have made comparison between groups problematic by inflating the response rate within this group. The following negative exponential function was then fitted to the mean response rates per condition: *y* = −a*exp(x*b); with *y* being the response rate and *x* being the trial number. The predicted peak response rate (*a*) and decay rate parameter (*b*) were extracted and analysed across conditions. The predicted peak response rate, the estimated point at which the function crosses the *x*-axis, is believed to provide a measure of the maximal motoric output of an animal. The decay rate has been proposed to reflect the effect of reinforcers upon subsequent bouts of responding, whereby a slower rate of decay in responding reflects an increased excitatory influence of rewards on subsequent behaviour (Phillips et al. 2017). The decay rate parameter has also been proposed to provide a measure of the rate of instrumental extinction (Simpson et al. 2011). Additional measures of motoric activity included the mean reward collection latency, the rate of IR beam breaks (beam breaks/sec), the rate of non-stimulus (blank) touchscreen responses (blank touches/sec) and the rate of magazine entries (magazine entries/sec)

### Statistical analysis

Analysis was conducted in SPSS Version 23 (IBM, Armonk, NY, USA) and the R software package (R Core Team 2017). Graphs were produced using Prism (GraphPad, La Jolla, CA, USA) and the ggplot2 package in R (Wickham 2009). To compare the effects of schedule at baseline, independent *t* tests were used. Levene’s test for equality of variance was employed and corrected where appropriate. For all other tests, repeated measures ANOVAs were employed. The Greenhouse-Geisser correction was applied for any violations of sphericity. All reported post hoc testing was adjusted using the Bonferroni correction for multiple comparisons.

## Results

### Effect of reinforcement schedule on baseline PR performance

**Fig. 1:**
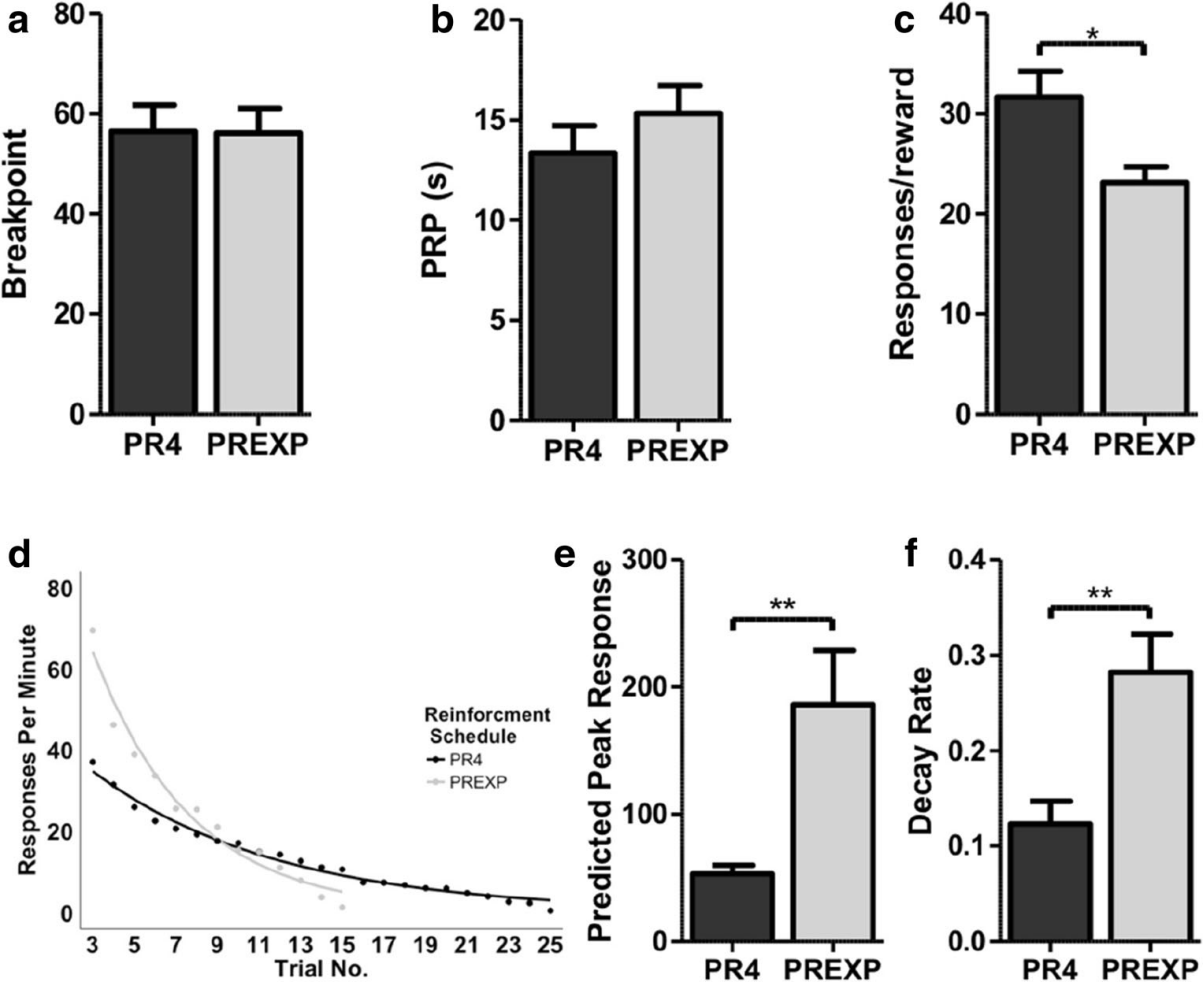
Effects of schedule of reinforcement on PR performance. **a** The mean breakpoint for both schedule groups. **b** The duration of the post reinforcement pause (PRP). **c** The mean number of touchscreen responses made per reward was higher in animals reinforced with the PR4 schedule. **d** The group mean response rate for each trial, from the third trial onwards for both reinforcement schedule. **e** Reinforcing animals under a PREXP schedule significantly increases the predicted peak response rate. **f** Reinforcing rats under a PREXP schedule significantly increases the rate of decay in responding. Error bars represent ±SEM. *p < .05; **p < .01

All measures were collapsed across five PR sessions. The mean breakpoint did not differ significantly according to schedule group (*t*(22) = .051, *p* = .96; Fig. 1a). The mean duration of the PRP also did not differ across reinforcement schedule groups (*t*(22) = 1.024, *p* = .317; Fig. 1b). The difference in the number of trials completed (and therefore rewards earned) did not reach statistical significance (*t*(22) *=* 1.982, *p* = .060). Animals reinforced under the PR4 schedule did, overall, make significantly more touchscreen responses in total for each reward earned (*t*(22) = 2.785, *p* < .05; Fig. 1c). There were no differences between the mean number of IR beam breaks made per second (*t*(22) = 1.441, *p* = .164. Response rates appeared to differ between schedule groups (Fig. 1d). The predicted peak response rate was significantly higher in animals reinforced under the PREXP schedule (*t*(22) = 3.067, *p <* .01; Fig. 1e). The response rate decay was also significantly greater in rats tested under the PREXP schedule of reinforcement (*t*(22) = 3.177, *p <* .01; Fig. 1f). Supplementary measures of motoric activity are available in Table 1.

**Table 1:** Mean values ± SEM of additional measures activity for both schedule types, as well as the number of rats in each condition that completed the 45-min session without emitting a response for 180 s. Additional motoric measures are of the reward collection latencies, rate of magazine entries (magazine entries per second), and the rate of nontarget (blank) screen responses (nontarget responses/sec) for all experimental conditions. Italic type signifies significant effects

	Reward collection latency		Magazine entries/sec		Nontarget responses/sec		No. of 45-min terminations	
	PR4	PREXP	PR4	PREXP	PR4	PREXP	PR4	PREXP
Baseline	*1.20* ± 0.04*	*1.58* ± 0.11*	0.08 ± 0.01	0.07 ± 0.01	0.03 ± 0.00	0.04 ± 0.01	33	14
Reward magnitude								
1 pell	1.11 ± 0.06	*1.36* ± *0.09*^*#*^	*0.07* ± *0.01*^*#*^	*0.05* ± *0.00*^*#*^	0.02 ± 0.00	0.03 ± 0.01	5	2
3 pellets	0.97 ± 0.08	*0.94* ± *0.12*^*#*^	*0.10* ± *0.01*^*#*^	*0.08 ± 0.01* ^*#*^	0.02 ± 0.00	0.03 ± 0.01	8	6
Prefeeding								
No feed	1.39 ± 0.09	1.51 ± 0.13	0.06 ± 0.01*	0.05 ± 0.00*	0.02 ± 0.00	0.02 ± 0.00	3	2
Prefeed	1.27 ± 0.05	1.56 ± 0.10	0.06 ± 0.01	0.04 ± 0.00	0.02 ± 0.00	0.03 ± 0.01	1	0
Raclopride								
Vehicle	1.23 ± 0.09*	1.63 ± 0.15*	0.08 ± 0.01*	0.06 ± 0.01*	0.02 ± 0.00	0.03 ± 0.01	0	0
0.03 mg/kg	1.28 ± 0.06	1.58 ± 0.14	0.07 ± 0.01	0.05 ± 0.01	0.03 ± 0.01	0.04 ± 0.02	2	2
0.3 mg/kg	1.44 ± 0.19*	2.65 ± 0.72*	*0.05 ± 0.01* ^*†*^	0.04 ± 0.00	*0.01 ± 0.00* ^*†*^	0.02 ± 0.01	1	0
Amphetamine								
Vehicle	1.44 ± 0.09*	1.80 ± 0.12*	0.09 ± 0.01	0.06 ± 0.01	0.04 ± 0.01	0.03 ± 0.01	1	0
0.1 mg/kg	1.40 ± 0.08*	1.71 ± 0.16*	0.08 ± 0.01	0.06 ± 0.01	0.03 ± 0.01	0.03 ± 0.01	1	2
1 mg/kg	1.35 ± 0.06	1.56 ± 0.09	0.10 ± 0.01	*0.09 ± 0.01* ^*†*^	0.05 ± 0.01	0.05 ± 0.01	6	8

*A significant group difference between schedule types, *p* < .05. ^#^A significant effect of increasing the reward magnitude, *p* < .05. ^†^A significant effect relative to the vehicle condition *p* < .05

### Increasing the magnitude of the reward enhances PR performance

**Fig. 2:**
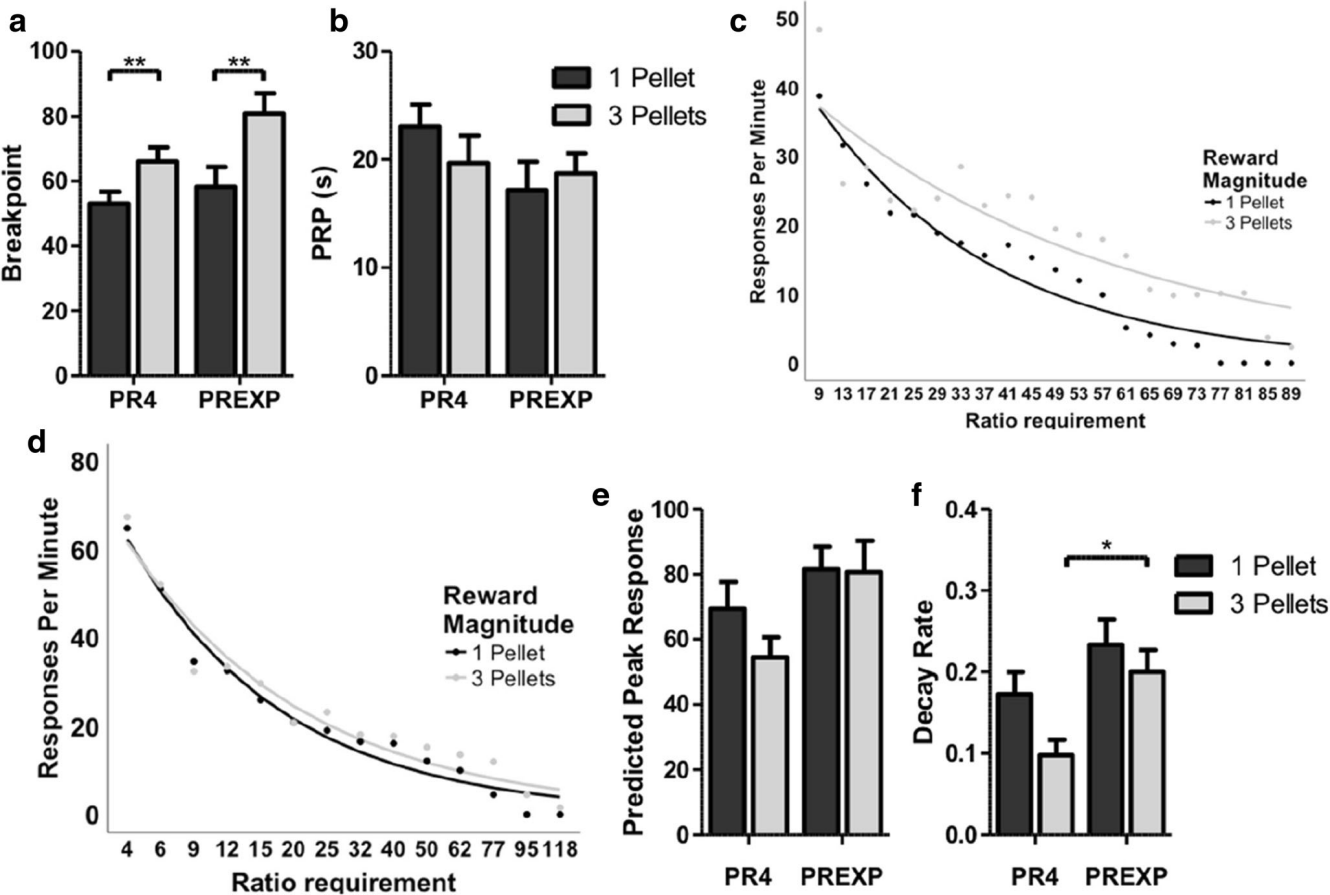
Increasing the magnitude of reward facilitates PR performance. **a** Reinforcing PR performance with three-pellet rewards significantly increases breakpoints in both schedule groups. **b** Changing the magnitude of reward does not alter the post reinforcement pause (PRP). **c** The PR4 group mean response rate for each trial, from the third trial onwards. **d** The PREXP group mean response rate for each trial, from the third trial onwards. **e** Increasing reward magnitude does not affect the predicted peak response rate. **f** Increasing the magnitude of reward does not affect the decay in responding. The PREXP group shows a greater decay rate when reinforced with three-pellet rewards. Error bars represent ±SEM. *p < .05; **p < .01

Increasing the magnitude of reward significantly increased breakpoint (*F*(1,22) = 35.183, *p* < .001; partial eta squared = .615; Fig. 2a). Post hoc testing revealed that breakpoints were significantly higher following three-pellet rewards in both schedule groups (both *p* < .01). Breakpoints were not affected by either schedule type or by any interaction between reward magnitude and schedule (both *p >* .05). There were no significant effects of reward magnitude, schedule type or interaction between the two upon either post reinforcement pausing (Fig. 2b) or the rate of IR beam breaks (all *p* > .05). Increasing reward magnitude also did not affect any additional measure of activity (Table 1).

Changing the magnitude of reward had did not affect response rates in either schedule group (Fig. 2c, d). The predicted peak response rate was not affected by either the reward magnitude, schedule type or any interaction between the two (all *p >* .05; Fig. 2e). The rate of decay in responding was grater in rats reinforced under the PREXP schedule (*F*(1,22) = 9.494, *p* < .01; partial eta squared = .301; Fig. 2f). Post hoc testing revealed that the decay in responding was higher in the PREXP when reinforced with three-pellet rewards (*p* < .05). The rate of decay in responding was not affected by either increasing the magnitude of rewards or by any interaction between reward magnitude and schedule type (*p* > .05).

### Prefeeding with chow prior to testing reduces effort expenditure

**Fig. 3:**
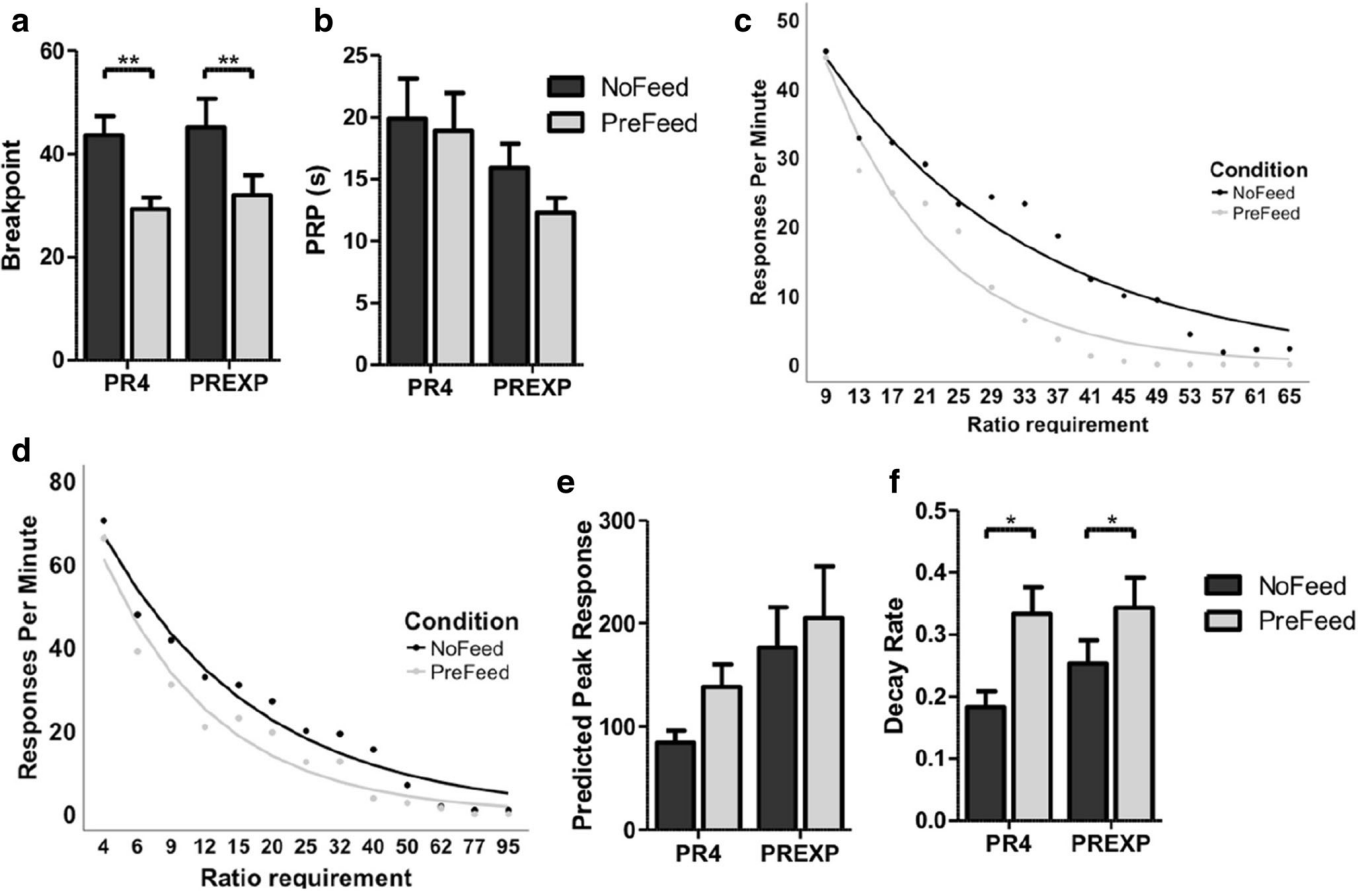
PR performance is supressed by prefeeding rats with homecage chow prior to testing. **a** Breakpoints are significantly lowered by prefeeding in both schedule groups. **b** Prefeeding with lab chow does not affect the duration of the mean post reinforcement pause (PRP). **c** The influence of prefeeding on the PR4 group mean response rate for each trial, from the third trial onwards. **d** The influence of prefeeding on the PREXP group mean response rate for each trial, from the third trial onwards. **e** Prefeeding does not affect the predicted peak response rate. **f** The decay rate was significantly increased after prefeeding with chow. Error bars represent ± SEM. *p < .05; **p < .01

Prefeeding the rats with chow significantly reduced breakpoints (*F*(1,22) = 22,796, *p* < .001, partial eta squared = .509; Fig. 3a). Breakpoints were significantly lower following prefeeding in both PR4 and PREXP schedule groups (both *p* < .01). Breakpoints were not significantly affected by either schedule of reinforcement or any interaction between schedule and prefeeding state (both *p* > .05). The duration of PRPs was significantly affected by reinforcement schedule type (*F*(1,22) = 4.494, *p* < .05, partial eta squared = .170); however, no effect survived multiple comparison adjustments in post hoc testing. The duration of the PRPs were not influenced by either prefeeding state or any interaction between prefeeding state and schedule type (both *p* > .05; Fig. 3b). There were no significant effects on the rate of IR beam breaks (all *p* > .05). Similarly, prefeeding had little effect on motoric activity (Table 1).

The change in response rates following prefeeding were analysed (Fig. 3c, d). The peak response rate was not significantly affected by either prefeeding state, schedule type or any interaction between the two (all *p* > .05, Fig. 3e). The rate of decay in responding was, however, significantly increased by prefeeding (*F*(1,22) = 9.839, *p* < .01; Fig. 3f). Post hoc testing revealed a significant increase in the rate of decay of responding in both schedule groups following prefeeding (both *p < .*05). The rate of decay was not significantly affected by either reinforcement schedule or by any interaction between prefeeding state and reinforcement schedule both (*p* > .05).

### Systemic administration of the D2/D3 receptor antagonist raclopride impairs PR performance

**Fig. 4:**
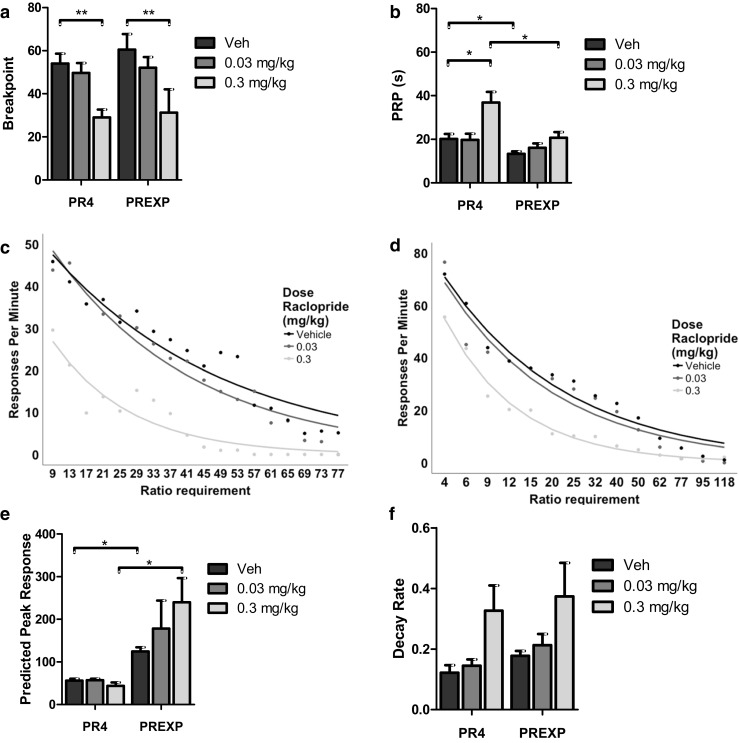
Systemic administration of raclopride disrupts PR performance. **a** Raclopride administered at a dose of 0.3 mg/kg significantly disrupts breakpoints reinforced under both PR4 and PREXP schedules. **b** 0.3 mg/kg raclopride significantly increases post reinforcement pauses (PRPs) in the PR4 condition only. The duration of PRPs was also significantly higher in the PR4 condition. **c** Suppression of response rates by raclopride in the PR4 group for each trial, from the third trial onwards. **d** PREXP group mean response rates are suppressed following raclopride administration. **e** Raclopride administration does not significantly affect the predicted peak response rate. Rats reinforced with the PREXP schedule are estimated to have a significantly higher peak response rate. **f** Raclopride administration did not significantly affect the decay rate. Error bars represent ± SEM. *p < .05; **p < .01

Two rats did not make any touchscreen responses following administration of 0.3 mg/kg raclopride; therefore, the data from these animals were removed from all raclopride analyses. Administration of raclopride significantly reduced breakpoints (*F*(2,40) = 14.113, *p* < .001; partial eta squared = .414; Fig. 4a). Breakpoints were significantly reduced by administration of 0.3 mg/kg compared to vehicle in both schedule groups (*p* < .01). Breakpoints were not significantly affected by either reinforcement schedule or by any interaction between schedule type and raclopride administration (both *p* > .05). The length of PRPs were significantly increased by raclopride administration (*F*(1.498,32.962) = 8.955, *p* < .01; partial eta squared = .289; Fig. 4b). Post hoc testing suggested that raclopride significantly increased pausing following administration of 0.3 mg/kg in the PR4 group (*p* < .05) but not the PREXP group. There was also a significant interaction between the dose of raclopride and reinforcement schedule (*F*(1.498,32.962) = 5.042, *p* < .05; partial eta squared = .186), suggesting that raclopride produced greater effects on pausing in animals reinforced under the PR4 schedule. PRPs were also significantly affected by schedule type (*F*(1,20) = 12.523, *p* < .01); partial eta squared = .363). Post hoc testing revealed that the mean PRP was significantly greater in the PR4 group following administration of both vehicle and 0.3 mg/kg raclopride. Raclopride administration significantly reduced the rate of IR beam breaks (*F*(1.309,26.185) = 6.298, *p* < .01*;* partial eta squared = .239). Post hoc tests revealed that 0.3 mg/kg raclopride reduced the rate of beam breaks, relative to administration of 0.03 mg/kg raclopride, in the PREXP group only (*p* < .05). The rate of IR beam breaks was not significantly affected by schedule type or by any interaction between raclopride and schedule type (both *p* > .05). Additional measures of motoric activity was largely unaffected by either dose of raclopride (Table 1).

Response rates following raclopride administration were analysed (Fig. 4c, d). The predicted peak response rate was significantly affected by schedule type (*F*(1,20) = 15.662, *p* < .01; partial eta squared = .439; Fig. 4e). Post hoc testing revealed that the PREXP group had a significantly higher predicted peak response rate following administration of vehicle and 0.3 mg/kg raclopride. The peak response rate was not affected by either raclopride administration or any interaction between raclopride and schedule type (both *p* > .05). Administration of raclopride did, however, significantly affect the decay in response rates (*F*(1.207, 24.142) = 5.860, *p* < .01; partial eta squared = .227; Fig. 4f). However, post hoc testing did not reveal any significant differences between doses. The decay in response rates was not significantly affected by either schedule type or by any interaction between schedule and raclopride administration (both *p* > .05).

### Systemic d-amphetamine facilitates PR performance

**Fig. 5:**
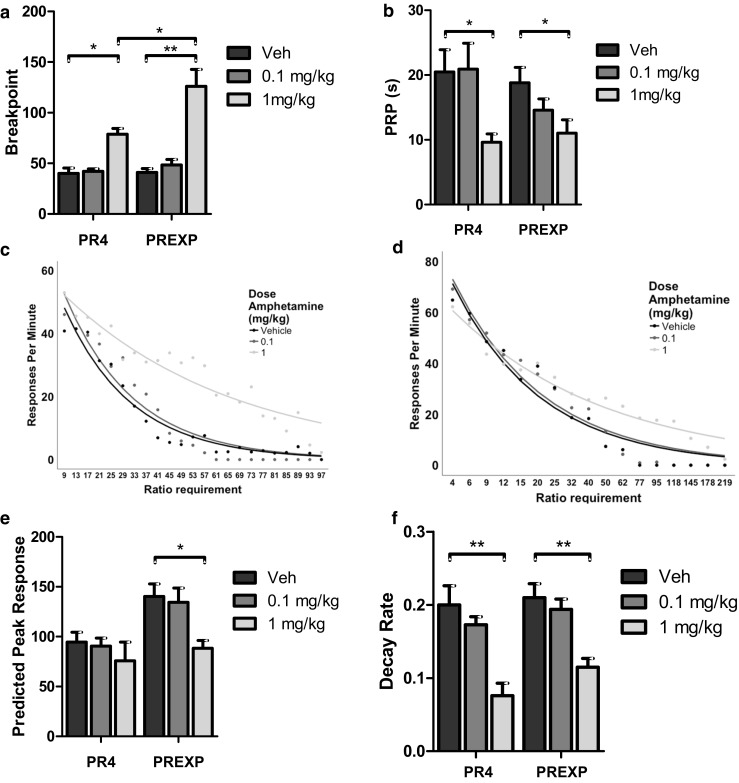
Facilitation of PR performance following systemic administration of d-amphetamine. **a** Administration of 1 mg/kg d-amphetamine significantly increases breakpoints in both schedule groups. Breakpoints are significantly higher in the PREXP group relative to rats reinforced under the PR4 schedule following administration of 1 mg/kg amphetamine. **b** The duration of the mean post reinforcement pause (PRP) is significantly reduced by 1 mg/kg amphetamine, in both reinforcement schedule conditions. **c** Enhancement of response rates following administration of amphetamine in rats reinforced with the PR4 schedule. **d** Response rates are enhanced following administration of amphetamine in rats reinforced with the PREXP schedule. **e** Amphetamine significantly reduces the predicted peak response rate in animals reinforced under the PREXP schedule only. The decay rate of responding is significantly reduced in rats in both schedule groups. Error bars represent ±1 SEM. *p < .05; **p < .01

Amphetamine administration significantly increased breakpoints (*F*(1.169,25.711) = 47.935, *p* < .001; partial eta squared = .685; Fig. 5a). Breakpoints were significantly greater in animals reinforced upon the PREXP schedule (*F*(1,22) = 5.072, *p* < .05; partial eta squared = .187). There was also a significant interaction between amphetamine and schedule type upon breakpoint (*F*(2,44) = 6.488, *p* < .01). Post hoc testing suggested 1 mg/kg amphetamine significantly increased breakpoint compared to vehicle for animals on both PREXP and PR4 schedules of reinforcement (both *p <* .05). However, breakpoints were significantly higher following administration of 1 mg/kg of amphetamine in the PREXP group. This finding indicates that amphetamine produced a greater effect upon breakpoints in animals tested on an exponential schedule of reinforcement compared to those on a linear reinforcement schedule. Amphetamine also had a significant effect on the mean PRP duration (*F*(2,44) = 13.451, *p* < .001; partial eta squared = .379; Fig. 5b). Post hoc testing revealed that 1 mg/kg amphetamine reduced the duration of PRPs relative to vehicle in both schedule groups. PRPs were not significantly affected by either schedule type or by any interaction between amphetamine and schedule (both *p* > .05). Amphetamine administration significantly increased the rate of IR beam breaks (*F*(1.440, 31.673) = 38.390, *p* < .001; partial eta squared = .636). Post hoc testing revealed that 1 mg/kg amphetamine increased the rate of beam breaks in both schedule groups relative to vehicle (*p <* .01). The rate of IR beam breaks was not significantly affected by either schedule or by any amphetamine × schedule interaction (both *p* > .05). In addition, amphetamine had little effect on any of the supplementary measures of motoric activity (Table 1).

Systemic administration of amphetamine appeared to enhance response rates (Fig. 5c, d). Amphetamine administration decreased the predicted peak response rate (*F*(2,44) = 6.237, *p* < .01; partial eta squared = .221; Fig. 5e). The predicted peak rate was reduced following 1 mg/kg amphetamine relative to all other doses (*p* < .05) in the PREXP group only. The predicted peak response rate was again significantly affected by schedule type (*F*(1,22) = 7.433, *p* < .05; partial eta squared = .253). Post hoc testing revealed that the peak rate was significantly higher in the PREXP group following administration of vehicle and 0.1 mg/kg amphetamine (*p* < .05). There was no significant interaction between amphetamine and reinforcement schedule (*p* > .05)*.* The rate of decay in responding was significantly reduced by amphetamine administration (*F*(2,44) = 25.548, *p* < .001; partial eta squared = .537; Fig. 5f). Post hoc testing revealed that 1 mg/kg amphetamine reduced the rate of decay relative to all other doses for both schedule groups (*p* < .01). The rate of decay in responding was not significantly affected by either schedule type or by any interaction between amphetamine and schedule (both *p* > .05).

## Discussion

Touchscreen versions of PR have been developed to assess motivation in mice (Heath et al. 2015), humans (Bland et al. 2016) and nonhuman primates (Weed et al. 1999). Maintaining high face validity between species may increase the likelihood of successful translation of findings (Bussey et al. 2012). Additionally, development of a rat touchscreen variant of progressive ratio will allow for assessment of motivation in this species within the same environment and using the same reinforcers earned in the assessment of more complex behaviours (Horner et al. 2013; Mar et al. 2013; Oomen et al. 2013). In the present study, a novel rat touchscreen PR task was assessed and found to be sufficiently sensitive for detecting changes in performance following outcome manipulations and systemic administration of dopaminergic drugs previously found to be efficacious in non-touchscreen versions of the schedule (e.g. Poncelet et al. 1983; Skjoldager et al. 1993; Cheeta et al. 1995; Schmelzeis and Mittleman 1996). The similarity in results across these different procedures further strengthens the use of measurement of responding under PR schedules of reinforcement to assay motivation. Furthermore, this represents the successful validation of the task for use in the rat touchscreen operant response system.

### Responding under a progressive ratio schedule of reinforcement as an assay of motivation

PR schedules are widely used, across species, to probe motivated behaviour. In spite of their common usage, PR schedules have a number of limitations, which have previously noted (Stewart 1975; Richardson and Roberts 1996; Killeen et al. 2009). Breakpoint is an unspecific measure and could reflect non-motivational changes in behaviour. Additionally, PR schedules can vary substantially in how the schedule of reinforcement progresses. As a consequence, it is not clear whether it is appropriate to compare PR performance between studies. The present study addresses some of these concerns. Firstly, we examined the dynamics of within-session changes in behaviour as a complementary measure to breakpoint. Specifically, we analysed the peak response rate as a measure of the initial motoric output and the rate of decay as an index of the motivational effects of reinforcers upon subsequent bouts of behaviour. Additionally, we compared behavioural performance between two markedly different schedules of reinforcement. The purpose of this was to see if, in otherwise equally motivated rats, interventions produced comparable effects on behaviour reinforced by different PR schedules. As behaviour was largely equally affected, it strengthens the case for results to be compared between studies that use different parameters.

An additional concern is that the decrease in responding towards the end of session could reflect a progressive satiation, rather than a reflection of the increasing effort costs (Hodos and Kalman 1963). Presently, the reward magnitude manipulation also suggested that progressive satiation was not affecting performance. Increasing the magnitude of rewards has been reported to affect breakpoints in an ‘inverted U’ fashion, with an initial facilitation in PR performance before decreasing breakpoints as animals become satiated (Hodos and Kalman 1963). As increasing the reward magnitude increased breakpoints, it suggests that rats had not yet reached the point where progressive satiation had begun to affect performance.

### Effect of reinforcement schedule on PR performance

Both linear and exponential schedules of reinforcement are widely used in PR tasks. The reinforcement schedule determines the number of operant responses required for each reward. Relative to the linear PR4 schedule, the PREXP schedule has an initially low response requirement for reinforcement which increases rapidly in subsequent trials. In the absence of any additional manipulations, breakpoints were remarkably similar between the two schedules (Fig. 1a). This is in spite of the difference in the total number of screen responses needed to achieve these breakpoints (Fig. 1c). Although, this finding may not generalise to every PR schedule, it indicates that prior history of reinforcement (at least within a session) is not the primary determinant of breakpoint. However, we did observe differences in the pattern of response rates between schedules. The initial predicted peak response rate was significantly higher in the PREXP schedule. Furthermore, rats reinforced under the PREXP schedule also displayed a significantly greater rate of decay in responding. The group differences observed are likely a reflection of the lower work requirements in the first few analysed trials in the PREXP condition, before a rapid increase in ratio requirements. Both the predicted peak response rate and decay rate appear independent of breakpoint. Examination of both whole and within-session measures may help to better understand motivational states of organisms during PR performance.

### Outcome manipulations

Increasing the magnitude of rewards resulted in a significant increase in breakpoints, in line with previous reports (Skjoldager et al. 1993; Eagle et al. 1999; Rickard et al. 2009). Larger magnitude rewards increase the vigour of operant responding (Skjoldager et al. 1993). This greater behavioural activation allows organisms to overcome greater effort costs to obtain rewards, resulting in higher breakpoints. Breakpoints may represent the outcome of a cost/benefit decision making process (Salamone et al. 2009). If an action or series of actions lead to a greater benefit (e.g. a larger food reward), then an organism should be more willing to overcome greater costs to obtain the goal. The rat touchscreen PR task was also sensitive to the effects of outcome devaluation through prefeeding. This is also in line with previous reports showing that inducing both specific (Skjoldager et al. 1993) and nonspecific satiety (Eagle et al. 1999) results in a reduction in breakpoints. Prefeeding with chow would be expected to devalue the reinforcer and reduce the effort an organism is willing to expend to receive the reward.

The length of PRPs was not significantly affected either by changing reward magnitudes or prefeeding. PRPs increase with the ratio requirements (Powell 1969; Baron et al. 1992). Increasing reward magnitudes increases trial completion and therefore the average ratio requirement within a session. This would be expected to increase the length of the average PRP. This result may explain why, overall, larger magnitude rewards did not decrease pausing. PRPs were also unaffected by prefeeding rats with homecage chow. This matches previous findings (Skjoldager et al. 1993; Eagle et al. 1999) of prefeeding on pausing under PR schedules. This is in contrast to the effects observed under FR schedules, where prefeeding animals has been reported to increase the duration of PRPs (Sidman and Stebbins 1954). Again, this may be as a result of prefeeding decreasing the total number of trials completed, and therefore decreasing the mean ratio requirement in these sessions. Together, this highlights a potential confound in evaluating performance based upon mean PRP across a PR session, without controlling for the total number of trials completed.

Neither increasing the reward magnitude nor prefeeding significantly altered the peak response rate. This is in agreement with the view that this variable reflects some measure of maximal motoric output (Phillips et al. 2017). Increasing the magnitude of reward also did not significantly affect the rate of decay in touchscreen responding. Previous reports suggest the efficacy of different food reinforcers in supporting PR performance does not appear to affect response rate decay (Kim et al. 2017). Therefore, it is not surprising that larger magnitude rewards do not affect the rate of decay, in spite of larger rewards supporting higher breakpoints. This further supports the hypothesis that the rate of decay reflects the qualitative effects of reinforcers upon behaviour, rather than a measure of behavioural activation. In contrast, reward devaluation through prefeeding significantly increased the rate of decay of responding. It is likely, therefore, that each food reward earned has a reduced ability to activate and support subsequent effortful behaviour resulting in an accelerated decay in response rates.

### Dopaminergic manipulations

Effort-based responding is highly sensitive to dopaminergic manipulations (Salamone and Correa 2012). Presently, systemic administration of raclopride and amphetamine increased and decreased breakpoints, respectively. This is in line with previous reports in lever-based versions of PR (Poncelet et al. 1983; Cheeta et al. 1995; Aberman et al. 1998), as well as in the mouse touchscreen version (Heath et al. 2015). It should be noted that as two rats failed to produce any touchscreen responses following the high dose of raclopride, it is possible that this dose also produced additional non-motivational effects such as impairing motoric function, in these rats. Additionally, in the remaining rats, the reward collection latency was increased (Table 1). However, the initial rate of responding was intact in these rats (Fig. 4e), suggesting the effects of raclopride upon PR performance were unlikely entirely a consequence of motoric disruption.

Amphetamine significantly increased breakpoints on both schedule types. However, amphetamine was able to produce a greater effect on breakpoints in animals reinforced under the PREXP schedule of reinforcement (Fig. 5a). This suggests that this schedule may have higher sensitivity, to detect changes in breakpoint, than the linear schedule employed in this study. Exponential PR schedules are commonly used in drug self-administration studies (Richardson and Roberts 1996). The rapidly increasing response requirement in later trials reduces the risk of ceiling effects in time-limited sessions (Roberts et al. 1989). In a similar vein, exponential schedules allow higher breakpoints to be reached with fewer responses and fewer rewards earned. This may reduce the influence of motor fatigue and/or satiety affecting the enhancement of breakpoints. It is unclear whether the present results would generalise to different linear PR schedules of reinforcement, but does suggest that certain reinforcement schedules can have differential sensitivity to detecting enhancements in motivated behaviour.

Both raclopride and amphetamine affected the duration of the PRPs. Amphetamine has previously been reported to decrease the length of PRPs (Evenden and Robbins 1983), whereas D2 receptor antagonists appear to increase pausing (Salamone 1986). The effects of dopaminergic compounds on PRP were in contrast to the lack of effects observed following the outcome modulations. The magnitude of the effects produced by the higher doses of raclopride and amphetamine appeared far larger than those produced by prefeeding and increasing reward magnitude. It may be the case that PRP as a measure is not as sensitive to changes in motivated behaviour as breakpoint, and larger effects are needed to detect significant changes in this measure. The present effects of amphetamine and raclopride upon PRP were not observed in the mouse touchscreen version of PR (Heath et al. 2015). Similarly, in this study, amphetamine had marked effects upon nonspecific locomotor activity in rats, but no effects were detected in mice performing the analogous task in a prior study (Heath et al. 2015). Species differences between mice and rats have been observed in a number of behavioural assays (see Young et al. 2013 for a review). The present results suggest an increased sensitivity to dopaminergic drugs in rats, relative to mice at equivalent doses. Few studies compare the two species, but there have been reports of differences in dopaminergic function in mice and rats under certain circumstances (e.g. Konstandi et al. 2000; Ralph-Williams et al. 2003).

Another notable result was the effect of amphetamine on the pattern of response rates. The high dose of amphetamine reduced both the peak response rate and the rate of decay in responding. The reduced initial peak rate may be a reflection of the anxiogenic and/or appetite supressing effects of amphetamine (MacPhail and Gollub 1974; Lapin 1993). The reduction in the rate of decay may have been a result of amphetamine altering the rats’ response to extinction. The low frequency of reinforcement relative to responding may result in extinction in later PR trials (Killeen et al. 2009). A slower decay in response rates may have reflected an increased resistance to extinction. However, if this were the case, it may have been expected that a greater effect upon the rate of decay would be observed in the PREXP group. The sharper increase in ratio requirements observed in the exponential schedule suggests a greater likelihood of extinction occurring relative to the linear schedule used in the PR4 group. As amphetamine reduced the rate of decay similarly in both groups, an increase in resistance to extinction is unlikely to be the sole explanation for a reduction in the rate of decay. A previous study, investigating within session changes in response rates reported that a similar dose of amphetamine (0.8 mg/kg), increased the activating or motivational effects of reinforcers upon behaviour (Mobini et al. 2000). The reduced rate of decay observed presently may reflect an increase in the behavioural activation following each reinforcer. As a consequence, each reinforcer is able to support behaviour for longer, which may also underlie, at least in part, the increased breakpoints following the high dose of amphetamine.

### Comparisons to other PR tasks

It is worth noting that, in the absence of any additional manipulations, breakpoints are lower in touchscreen PR than those observed in lever-press PR schedules. For example, across both linear and exponential schedule types, breakpoints in excess of 100 are typically observed in lever-responding rats (Skjoldager et al. 1993; Bezzina et al. 2015; Olarte-Sánchez et al. 2015). Therefore, the present findings of rats returning breakpoints in the region of ~ 45–55 are considerably lower than those seem with levers. The rate of operant responding is highly sensitive to physical characteristics such as the height of the lever (Skjoldager et al. 1993) and the required response force (Alling and Poling 1995). The touchscreen used in the present study use IR photocells to record screen touches (in fact, the rat is not strictly speaking required to ‘touch’ the screen). Therefore, touchscreen responding would be expected to require less physical effort than responding on a lever. The differences in breakpoint, therefore, cannot be explained in terms of force requirements. One possibility is that the biophysical feedback from touchscreen responding is considerably less than that obtained by pressing a lever. In turn, there may be less salient cues to associate with reward. Pavlovian cues associated with reward are able to strongly influence instrumental behaviour (Rescorla and Solomon 1967). The reduced salience of cues associated with touchscreen responding relative to lever pressing may therefore reduce their invigorating effects upon responding (e.g. Saunders and Robinson 2011).

A separate possibility is the delay between response and reward. Increasing the delay from a response to a reward will shift behaviour to obtaining an immediately available, but less preferred reward (Thiébot et al. 1985). In the current touchscreen PR task, it is only possible to make a response every 0.5 s. This is due to a brief stimulus offset, added to provide visual feedback that a response has been made (see Methods). As a consequence, the rate of responding would be expected to be lower than a lever-based version of PR where rats are able to make multiple lever responses every second (e.g. Olarte-Sánchez et al. 2015). The longer time taken to complete each ratio may increase the costs associated with obtaining reward and result in animals ceasing responding earlier. The reduced breakpoints and response rates in touchscreen PR may confer certain advantages: the avoidance of ceiling effects that may obscure potential faciliatory effects of interventions, particularly when using time-limited schedules, and a lower number of responses which may reduce the potentially confounding influences of satiation and motor fatigue upon performance.

Taken together, this study demonstrates the successful adaption and validation of progressive ratio for the rat operant touchscreen system. Like the mouse touchscreen- and traditional lever-based versions, the rat touchscreen PR variant is sufficiently sensitive to detect bidirectional changes in motivated behaviour following outcome manipulations and dopaminergic drugs. Furthermore, this study demonstrates that the use of exponential schedules of reinforcement may provide a greater sensitivity to detecting the effects of compounds that enhance PR performance. Additionally, this study demonstrates the utility of the complementary approach of studying within-session changes in behaviour in addition to cumulative parameters, such as breakpoint. Finally, effort-based motivated behaviour can now be assayed, with high face validity, across species.
